# Chk1 Inhibition as a novel therapeutic strategy for treating triple-negative breast and ovarian cancers

**DOI:** 10.1186/1471-2407-14-570

**Published:** 2014-08-07

**Authors:** Christopher Bryant, Rebecca Rawlinson, Andrew J Massey

**Affiliations:** Vernalis R&D Ltd, Granta Park, Cambridge, CB21 6GB UK

**Keywords:** Chk1, Breast cancer, Ovarian cancer, V158411, DNA repair, Triple-negative

## Abstract

**Background:**

Chk1 inhibitors are currently in clinical trials as putative potentiators of cytotoxic chemotherapy drugs. Chk1 inhibitors may exhibit single agent anti-tumor activity in cancers with underlying DNA repair, DNA damage response or DNA replication defects.

**Methods:**

Here we describe the cellular effects of the pharmacological inhibition of the checkpoint kinase Chk1 by the novel inhibitor V158411 in triple-negative breast cancer and ovarian cancer. Cytotoxicity, the effect on DNA damage response and cell cycle along with the ability to potentiate gemcitabine and cisplatin cytotoxicity in cultured cells was investigated. Western blotting of proteins involved in DNA repair, checkpoint activation, cell cycle and apoptosis was used to identify potential predictive biomarkers of Chk1 inhibitor sensitivity.

**Results:**

The Chk1 inhibitors V158411, PF-477736 and AZD7762 potently inhibited the proliferation of triple-negative breast cancer cells as well as ovarian cancer cells, and these cell lines were sensitive compared to ER positive breast and other solid cancer cells lines. Inhibition of Chk1 in these sensitive cell lines induced DNA damage and caspase-3/7 dependent apoptosis. Western blot profiling identified pChk1 (S296) as a predictive biomarker of Chk1 inhibitor sensitivity in ovarian and triple-negative breast cancer and pH2AX (S139) in luminal breast cancer.

**Conclusions:**

This finding suggests that Chk1 inhibitors either as single agents or in combination chemotherapy represents a viable therapeutic option for the treatment of triple-negative breast cancer. pChk1 (S296) tumor expression levels could serve as a useful biomarker to stratify patients who might benefit from Chk1 inhibitor therapy.

## Background

Breast cancer is the most common cancer affecting women and the second most common cause of death due to cancer [1]. This highly heterogeneous disease has been classified into various subgroups (namely luminal, HER2 positive, basal-like and normal breast) based on gene expression profiles and phenotypic characteristics [2]. Triple-negative breast cancer (TNBC) is characterized by a lack of expression of estrogen receptor (ER), progesterone receptor (PR) and ErbB-2/human epidermal growth factor receptor 2 (HER2) [3, 4]. TNBC shares many of the gene expression profiles and phenotypical features of basal-like breast cancer [5, 6]. The prognosis for breast cancer patients with basal-like disease, including TNBC, is generally much poorer than those of other sub-groups due to the aggressive nature of this sub-group. Whilst basal-like and triple-negative breast cancers have been demonstrated to be chemo-sensitive to neoadjuvant therapy, the relapse rates are more aggressive resulting in a worse overall survival [7, 8]. Unlike ER, PR or HER2 positive tumors where clear molecular targets for therapeutic intervention exist, identifying specific molecular targets for TNBC has proved more elusive.

Breast tumors arising from hereditary BRCA mutation carriers are mostly triple-negative and share a striking resemblance to sporadic basal-like and triple-negative breast cancers suggesting that TNBC might bear defects in DNA repair thereby endowing a degree of “BRCAness” on these cancers [9]. BRCA mutant cell lines have been shown to be sensitive to DNA damaging agents such as cisplatin [10] as well as inhibitors of the repair protein poly (ADP-ribose) polymerase (PARP), a key component of the base excision DNA repair pathway [11, 12]. Recent studies have shown that basal-like TNBC cell lines are also sensitive to PARP inhibitors and cytotoxic chemotherapy drugs such as gemcitabine and cisplatin [13, 14]. Inhibition of PARP in cancer cells with defects in DNA double strand break (DSB) repair pathways (such as BRCA mutant breast cancer) results in an accumulation of single strand breaks (SSBs) leading to replication fork collapse, DSB generation and cell death. This reliance on alternative DNA repair pathways may make cancers such as TNBC sensitive to other agents that target DNA damage response pathways.

The DNA damage signaling response pathway (DDR) is activated by DNA breaks induced by a variety of endogenous and external insults including therapies currently used for the treatment of cancer such as ionizing radiation and cytotoxic chemotherapeutic agents such as gemcitabine, irinotecan and cisplatin. Activation of the DDR results in a number of cellular responses including checkpoint activation and cell cycle arrest, initiation of DNA repair, regulation of transcription and apoptosis [15, 16]. The serine-threonine checkpoint kinases Chk1 and Chk2 are activated by the ATR and ATM kinases in response to DNA breaks and form the key link between the sensing kinases ATR/ATM and the cell cycle machinery [17]. Phosphorylation of Chk1 leads to checkpoint activation and cell cycle arrest at the G1/S, intra S or G2/M phases. Despite their similarity in name, Chk1 and Chk2 differ substantially in the structure of their kinase pocket [18, 19] and in their cellular function with Chk1 suggested to be the major component responsible for responses to DNA damage. Inhibiting Chk1 following genotoxic stress (such as that induced by cytotoxic chemotherapy) results in checkpoint abrogation, inhibition of DNA repair and induction of cell death in cells with a defective p53 response [20, 21]. Small molecule inhibitors of predominantly the Chk1 kinase have been readily sought as a mechanism through which the anti-tumor activity of cytotoxic chemotherapeutics may be increased whilst sparing normal cells [22, 23]. This approach is currently being tested in the clinic with a variety of agents including LY2603618 [24], GDC-0425, GDC-0575, MK-8776 [25], PF-477736 [26] and AZD7762 [27] in combination with a range of standard of care chemotherapy drugs.

It has also been postulated that DNA damage response checkpoints and especially Chk1 kinase activity may be critical for the normal replication of cancer cell lines with specific underlying defects in DNA repair or DNA damage response pathways, or DNA replication defects. For example, the Fanconi Anaemia (FA) repair pathway is responsible for repairing crosslinked DNA and maintaining chromosomal stability [28]. FA deficient cell lines were found to be sensitive to Chk1 silencing by siRNA compared to FA proficient cells [29]. We hypothesized that, given their likeness to DNA repair compromised hereditary BRCA mutated breast cancers, triple-negative breast cancer may be sensitive to cell killing by Chk1 inhibitors.

V158411 is a novel, potent, selective inhibitor of recombinant Chk1 and Chk2 kinases *in vitro* with IC_50_s of 3.5 and 2.5nM respectively [30]. In p53 defective HT29 cells, V158411 inhibited the etoposide induced auto-phosphorylation of Chk1 on Ser296 with an IC_50_ of 48 nM and Chk2 on Ser516 with an IC_50_ of 904 nM indicating a 19-fold cellular selectivity for Chk1 over Chk2. V158411 potentiated cytotoxic chemotherapy in p53 defective cancer cells *in vitro* and *in vivo.* We therefore evaluated the single agent cytotoxic potential of V158411 against a panel of solid cancer cell lines including those derived from breast and ovarian cancer. We further profiled the panel of cell lines to understand and identify potential biomarkers predictive of response to Chk1 inhibition. The data provides a preclinical rationale to support the clinical testing of Chk1 inhibitors as single agents and in combination with cytotoxic chemotherapy in patients with triple-negative breast cancer.

## Methods

### Cell culture and cytotoxicity assay

All cells were obtained from the American Type Culture Collection and cultured in DMEM, RPMI or McCoys 5a containing 10% FCS (Invitrogen). The cytotoxicity of V158411 was determined following exposure of cells in 96 well plates to a 10-point titration for 72 hours. Cell proliferation was determined using sulphorhodamine B (Sigma) staining following protein precipitation with 10% TCA. For cell counts, cells were seeded in 6 well plates and counted following trypsinisation after 72 hours using a haemocytometer with trypan blue staining.

### Compounds

V158411 was synthesized according to the method described in [30] and prepared as a 20 mM DMSO stock in DMSO. Solid stocks were purchased from the indicated suppliers and prepared as concentrated stock solutions in the appropriate solvent: gemcitabine (Apin Chemicals Inc), 20 mM in H_2_O; cisplatin (Selleckchem), 3.33 mM in 1% NaCl in H_2_O; oxaliplatin (Tocris), 5 mM in H_2_O; carboplatin (Tocris), 25 mM in H_2_O; PF-477736 (Selleckchem), 20 mM in DMSO and AZD7762 (Axon Medchem), 20 mM in DMSO.

### Determination of caspase-3/7 dependent apoptosis

Cells were seeded in 96 well plates and treated with 10-times the GI_50_ of V158411 for 24 or 48 hours. Caspase-3/7 activity was determined using a homogenous caspase-3/7 luminescence kit (Promega).

### Antibodies and western blotting

Anti-pHistone H3 (S10) was obtained from Millipore; Chk1, pChk1 (S317), pChk1 (S345), pChk2, pChk2 (T68), pCdc25c (S216), 53BP1, Cdc2, pCdc2 (Y15), Cyclin B1, D1 and E, PARP, pERK1/2, ERK 1/2, AKT, pAKT (S473), Bcl-X_L_, GAPDH and pH2AX (S139) from Cell Signaling Technologies; pChk1 (S296), FANCF and FANCD2 from Abcam, and Bcl-2 and Mcl-1 from Santa Cruz. Treated and untreated cells were washed once with PBS and lysed in RIPA buffer containing protease and phosphatase inhibitor cocktails (Roche). Protein concentration was determined using BCA kit (Pierce). Equal amounts of lysate were separated by SDS-PAGE and western blot analysis conducted using the antibodies indicated above.

### Flow cytometry

Cells were seeded in 6 well plates and subsequently treated with the indicated concentrations of V158411 for 24 or 48 hours. All cells were harvested, fixed in 70% ethanol and stained with propidium iodide/RNase A. Cell cycle profiles were examined by flow cytometry using a FACSArray cytometer (BD) and FACSDiva software (BD).

### Potentiation assays

5x10^3^ cells per well were seeded in 96 well plates and incubated overnight. Cells were treated with a 10-point titration of gemcitabine or cisplatin in the presence of a fixed concentration of V158411 for 72 hours. The effect on cell proliferation was determined using a CellTiter 96 AQ_ueous_ One Solution Cell Proliferation Assay (MTS, Promega).

### Ethical approval

None of the research in this manuscript involved human subjects, human material, or human data, or used regulated vertebrates or invertebrates.

## Results

### Pharmacological abrogation of Chk1 activity inhibits cell proliferation and induces caspase activation in human triple-negative breast and ovarian cancer cell lines

Sporadic basal-like or triple-negative breast cancers strongly resemble breast cancers originating from hereditary BRCA defective patients and may harbor BRCA-mimetic defects in DNA repair pathways. To test the hypothesis that triple-negative breast cancers are sensitive to Chk1 inhibitors in the absence of cytotoxic chemotherapy agents, a panel of sporadic triple-negative and luminal breast cancer cell lines [31] as well as several ovarian cancer cell lines were tested for their sensitivity to V158411 and compared against a panel of cell lines derived from cancers of the lung, colon or prostate.

**Figure 1:**
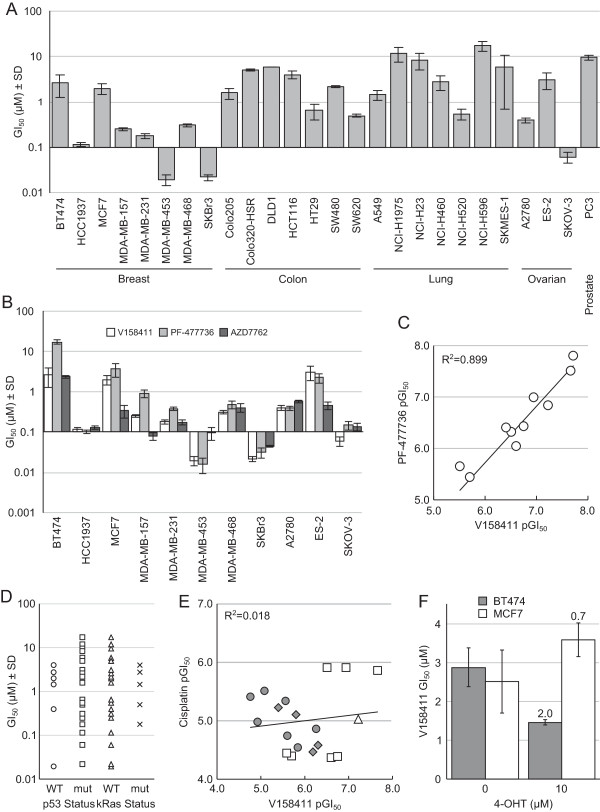
**Inhibition of Chk1 inhibits cell proliferation in human triple-negative breast and ovarian cancer cell lines. A**. Human tumor cell lines were treated with the Chk1 inhibitor V158411 for 72 hours and the cell number determined using the SRB assay. Values are the average of at least four independent determinations ± SD. **B**. Breast and ovarian cell lines were exposed to the Chk1 inhibitors V158411, PF-477736 and AZD7762 for 72 hours. Cell number was determined by SRB assay. Values are the mean ± SD for n ≥ 4. **C**. Comparison of cell line sensitivity to V158411 and PF-477736. **D**. Sensitivity to V158411 was independent of p53 mutational status. The p53 mutation status was determined from the Cancer Genome Project. **E**. Comparison of the sensitivity of breast (square), ovarian (triangle), colon (diamond) and lung (circle) cell lines to growth inhibition by V158411 and cisplatin. **F**. ER-positive breast cancer cells were exposed to V158411 in the presence of 0 or 10 μM 4-hyrdroxytamoxifen (4-OHT) for 72 hours and the cell number (GI_50_) determined by SRB assay. The values are the average of 4 determinations ± SD and the fold difference between 0 and 10 μM 4-OHT shown.

**Figure 2:**
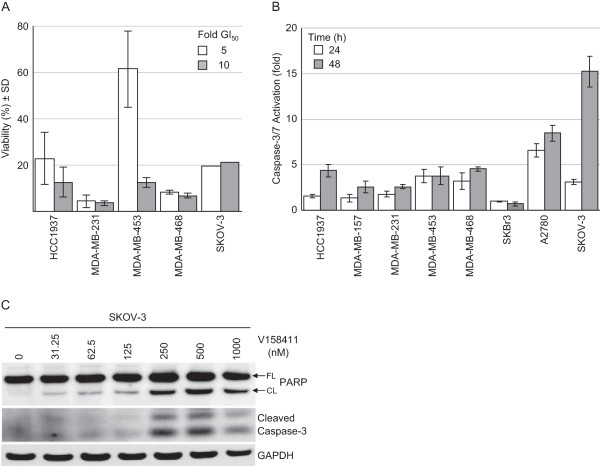
**V158411 reduces cell viability and induces caspase-3/7 dependent apoptosis. A**. V158411 inhibits the viability of triple-negative breast and ovarian carcinoma cells. Cells were exposed to 5 or 10-times the GI_50_ of V158411 for 72 hours and the cell viability determined using trypan blue staining. Values are the average of ≥4 determinations ± SD. **B**. Caspase-3/7 dependent apoptosis is induced in triple-negative breast and ovarian carcinoma cell lines *in vitro*. Cells were exposed to 10-times the GI_50_ of V158411 for 24 or 48 hours and caspase activity determined using a homogenous caspase-3/7 activation kit. Values are the average of 3 replicates ± SD. **C**. Caspase-3/7 activation was confirmed by immunoblotting in SKOV-3 cells treated for 48 hours with the indicated concentrations of V158411. FL, full length; CL, cleaved.

The TNBC cell lines exhibited increased sensitivity to V158411 compared to either the ER or HER2 positive breast cancer cell lines BT474 and MCF7 as well as a range of colon, lung and prostate cancer cell lines (Figure 1A and Table 1). The exception was the HER2 positive, ER negative breast cancer line SKBr3 which exhibited V158411 sensitivity equivalent to the TNBC cell lines. In addition, two out of the three ovarian cell lines tested exhibited increased sensitivity to V158411. We confirmed the sensitivity of TNBC and ovarian cancer cell lines to Chk1 inhibition with two additional Chk1 inhibitors PF-477736 [26] and AZD7762 [27]. As observed with V158411, the TNBC and ovarian cell lines exhibited increased sensitivity to these two agents compared to the MCF7 and BT474 breast cell lines (Figure 1B). There was a strong correlation between cell line sensitivity to V158411 and PF-477736 (R^2^ = 0.899, Figure 1C). The correlation between V158411 and AZD7762, and PF-477736 and AZD7762 sensitivity was less strong (R^2^ = 0.653 and 0.635 respectively) and may be due to the reduced kinase selectivity of AZD7762 compared to V158411 and PF-477736. Sensitivity to V158411 was independent of p53 or *kRas* mutational status (Figure 1D). Likewise, the increased sensitivity of the triple-negative breast and ovarian cancer cell lines to V158411 did not correlate with increased sensitivity to the DNA damaging agent cisplatin (R^2^ = 0.018, Figure 1E). We compared the sensitivity of cell lines to V158411 and a variety of DNA damaging drugs using data from the Genomics of Drug Sensitivity in Cancer data set (Release 4, [32]). Again, there was no correlation between sensitivity to V158411 and camptothecin, cytarabine, doxorubicin, etoposide, cisplatin, gemcitabine, olaparib or mitomycin C (R^2^ < 0.1). Inhibition of the estrogen receptor with 4-hydroxytamoxifen did not increase the sensitivity of the ER positive BT474 and MCF7 cell lines to Chk1 inhibition (Figure 1F). Treatment with V158411 led to a reduction in cell viability in sensitive TNBC and ovarian cell lines (Figure 2A). This reduction in viability was accompanied by V158411 induced caspase-3/7 dependent apoptosis in the sensitive cell lines (Figure 2B and C). The resistance of the BT474 and MCF7 cell lines appeared independent of p53 mutational status and ER or HER2 receptor expression status.

**Table 1:** **Breast and ovarian cell lines used in this study and their sensitivity to V158411**

Tissue	Cell line	Gene cluster	ER	PR	HER2	TP53	GI _50_(μM)
Breast	BT474	Lu	+	+	+	Mut	2.6 ± 1.3
	HCC1937	BaA	-	-		Mut	0.11 ± 0.01
	MCF7	Lu	+	+		WT	2.0 ± 0.5
	MDA-MB-157	BaB	-	-		Mut	0.25 ± 0.02
	MDA-MB-231	BaB	-	-		Mut	0.18 ± 0.02
	MDA-MB-453	Lu	-	-		WT	0.019 ± 0.005
	MDA-MB-468	BaA	-	-		Mut	0.31 ± 0.02
	SKBr3	Lu	-	-	+	Mut	0.022 ± 0.003
Ovarian	A2780		-		-	WT	0.39 ± 0.05
	ES-2					Mut	3.1 ± 1.2
	SKOV-3		-		+	Mut	0.06 ± 0.02

ER, estrogen receptor; PR, progesterone receptor; BaA, Basal A; BaB, Basal B; Lu, Luminal; WT, wild type; Mut, mutant; breast data from [31]. Values represent the mean ± SD of n ≥ 4.

### Chk1 inhibition induces Chk1 degradation and H2AX phosphorylation

**Figure 3:**
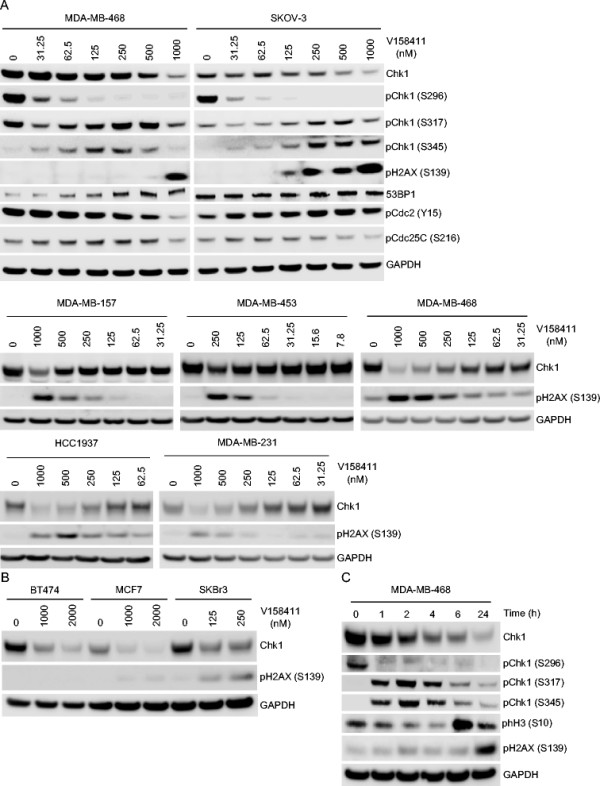
**Chk1 inhibition by V158411 induces Chk1 degradation and H2AX phosphorylation.** Triple-negative breast or ovarian cancer cells **(A)** or luminal breast cancer cell lines **(B)** were exposed to the indicated concentrations of V158411 for 24 hours. **C**. MDA-MB-468 TNBC cells were treated with 1 μM V158411 for 0 to 24 hours. Protein changes were assessed by immunoblotting.

The effect of V158411 on the Chk1 DNA damage response pathway in MDA-MB-468 and SKOV-3 cells was determined. Treatment of either cell line with V158411 for 24 hours resulted in decreased Chk1 autophosphorylation (on serine 296). This inhibition of Chk1 correlated with an activation of ATR and increased DNA strand breakage as measured by increased Chk1 phosphorylation on serine 317 and 345 and increased pH2AX (S139) (Figure 3A). Treatment of all five TNBC cell lines or the sensitive SKOV-3 ovarian cell line with V158411 for 24 hours led to a dose dependent decrease in Chk1 protein levels and a concomitant increase in the amount of H2AX phosphorylated at Ser139 (Figure 3A). The dose at which V158411 decreased Chk1 protein levels and increased H2AX phosphorylation correlated closely with the sensitivity of the cell line to V158411 toxicity. In comparison, in the two resistant luminal breast cancer cell lines BT474 and MCF7, treatment with an equitoxic dose of V158411 resulted in a decrease in Chk1 protein levels but not a subsequent increase in H2AX phosphorylation (Figure 3B). The response of the sensitive luminal breast cancer cell line SKBr3 mirrored that of the sensitive TNBC cell lines. A time course of V158411 treatment in MDA-MB-468 cells indicated that inhibition of Chk1 autophosphorylation (S296) occurred rapidly (in under 1 hour) and that activation of ATR (as measured by increased phosphorylation of Chk1 at S317/S345) was coincidental with inhibition of Chk1. Maximal Chk1 reduction and H2AX phosphorylation was delayed compared to Chk1 inhibition requiring 24 hours for the maximal response to be observed (Figure 3C).

### Chk1 inhibition induces cell cycle arrest and DNA fragmentation

**Figure 4:**
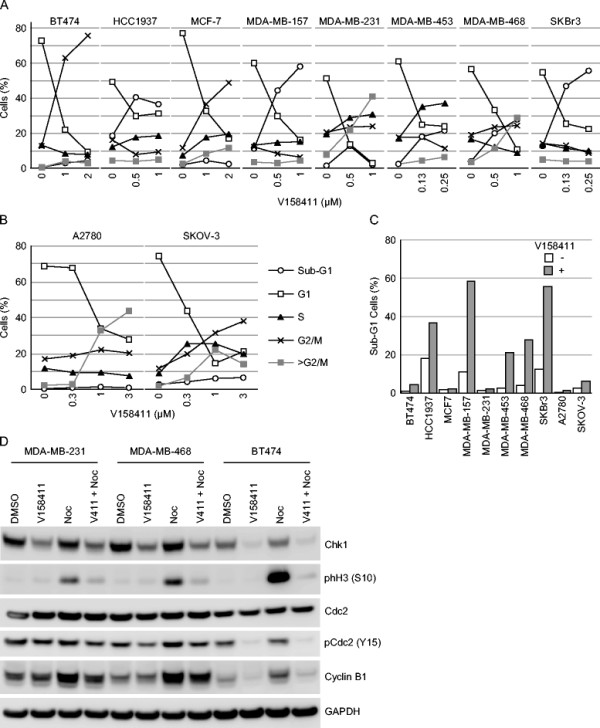
**Cell cycle changes associated with Chk1 inhibition in breast and ovarian cells.** Cell cycle profiles of TNBC **(A)** or ovarian cancer **(B)** cells were determined by PI staining following treatment with the indicated concentrations of V158411 for 24 hours. **C**. The fraction of cells with a sub-G1 DNA content was quantitated from the cell cycle profiles following 48 hour incubation. **D**. MDA-MB-231, MDA-MB-468 or BT474 cells were treated with 1 μM V158411 in the presence or absence of 0.5 μM nocodazole for 24 hours. Protein levels were subsequently assessed by western blotting.

Treatment of breast and ovarian cancer cell lines with V158411 lead to dramatic changes in the cell cycle distribution of the treated cells. In both sensitive and resistant cell lines, V158411 treatment massively decreased the fraction of cells in G1. The resistant luminal breast cancer lines BT474 and MCF7 responded by arresting in G2/M whilst in the sensitive TNBC and luminal SKBr3 cell lines, the decrease in G1 correlated with an increase in sub-G1 or greater than G2/M DNA content indicative of an increase in DNA fragmentation and chromosomal breakages (Figure 4A). In the two sensitive ovarian cell lines, V158411 again dramatically reduced the G1 fraction of cells and increased the fraction of cells with fragmented DNA (sub-G1 or > G2/M) (Figure 4B). Overall, the sensitive TNBC and SKBr3 cell lines exhibited the greatest increase in sub-G1 DNA content following V158411 treatment (Figure 4C). To evaluate if cells were progressing into mitosis and undergoing death via mitotic catastrophe, we utilized nocodazole to trap cells in mitosis following V158411 treatment. Nocodazole increased the fraction of MDA-MB-231, MDA-MB-468 and BT474 cells in mitosis as evidenced by an increase in the levels of phH3 (S10). However, treatment with V158411 plus nocodazole did not lead to an increase in the number of mitotic cells compared to V158411 alone (Figure 4D). In the resistant BT474 cells but not the two sensitive TNBC cell lines, V158411 treatment reduced the phosphorylation of Cdc2 on Tyr15 and is consistent with the G2/M arrest observed in this cell line.

### Western blot profiling of breast and ovarian cell lines identified Chk1 Ser296 phosphorylation as a predictive biomarker of sensitivity

**Figure 5:**
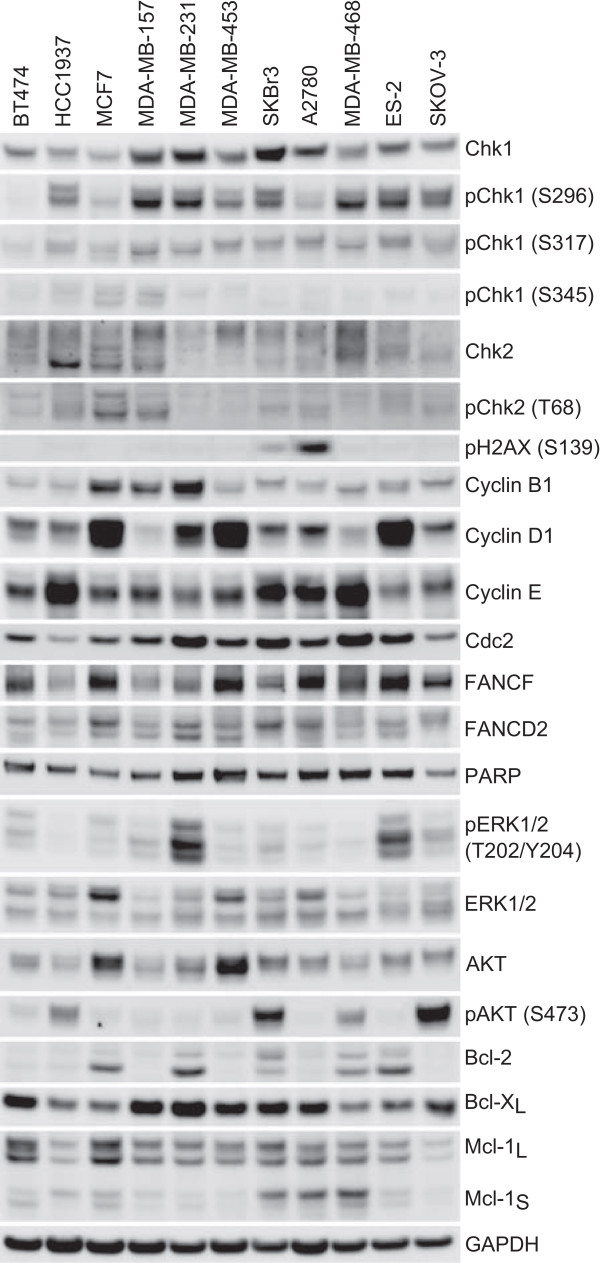
**Western blot analysis of breast and ovarian cell lines.** Untreated whole cell protein extracts were prepared from the indicated cell lines and the expression levels of various protein markers determined by western blotting.

Identifying biomarkers that potentially predict for sensitivity to single agent Chk1 inhibition is important for translating the therapy into the right patients in the clinic. We examined the expression levels of a variety of checkpoint, cell cycle, apoptosis and DNA repair associated proteins across the panel of sensitive and resistant ovarian cell lines. The expression levels of these proteins, following immunoblot analysis, is illustrated in Figure 5. Two markers were identified as correlating with increased sensitivity to V158411 cytotoxicity. All the TNBC cell lines exhibited high levels of Chk1 phosphorylated on Ser296 compared to the ER positive breast cancer cell lines. In the sensitive luminal breast cancer cell line SKBr3, the endogenous levels of H2AX phosphorylated on Ser139 was much higher compared to all other cell lines. In the ovarian cell lines, the sensitive A2780 and SKOV-3 as well as the resistant ES-2 cell line exhibited high levels of endogenous pChk1 (S296). Chk1 was expressed in variable amounts across all eleven cell lines examined whilst the levels of Chk1 phosphorylated on the ATM/ATR sites Ser317 and 345 was virtually undetectable. The increased levels of Chk1 (S296) and H2AX (S139) phosphorylation are consistent with underlying defects in DNA repair and/or replication. Analysis of other proteins associated with DNA replication or the DDR response did not identify a consistent mechanism for Chk1 activation.

### V158411 potentiates cytotoxic chemotherapy in TNBC and ovarian cancer cell lines

**Figure 6:**
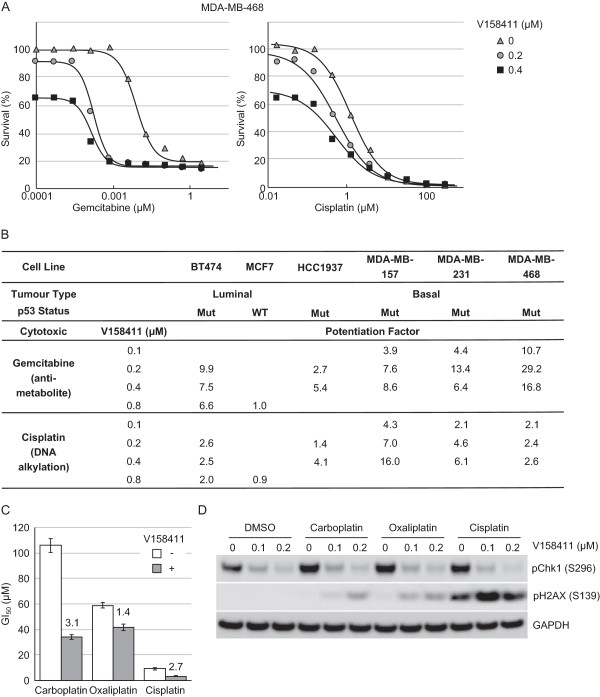
**V158411 potentiates the anti-tumor efficacy of cytotoxic chemotherapeutic drugs** ***in vitro.*** **A**. Curves representing the 72 hour antiproliferative effect of gemcitabine (left) or cisplatin (right) in MDA-MB-468 cells in combination with 0, 0.2 or 0.4 μM V158411. **B**. *In vitro* potentiation of gemcitabine and cisplatin cytotoxicity by 0.1, 0.2, 0.4 or 0.8 μM V158411 in p53-proficient or p53-deficient breast cancer cell lines. Potentiation factor was calculated by IC_50(cytotoxic agent alone)_/IC_50(combination treatment)_. **C**. *In vitro* potentiation of carboplatin, oxaliplatin and cisplatin cytotoxicity in SKOV-3 ovarian cancer cells by 0.1 μM V158411. The GI_50_ values are the average of 3 determinations ± SD with the potentiation factor highlighted above each bar. **D**. SKOV-3 cells were treated with 250 μM carboplatin, 80 μM oxaliplatin or 20 μM cisplatin for 24 hours in the presence of 0, 0.1 or 0.2 μM V158411 for 24 hours. The amount of Chk1 and H2AX phosphorylation was determined by western blotting.

The ability of V158411 to potentiate the cytotoxicity of a variety of cytotoxic chemotherapeutic drugs was assessed across a panel of luminal breast cancer and TNBC cell lines. V158411 effectively potentiated the growth inhibitory activity of gemcitabine and cisplatin in the panel of p53-defective but not p53-proficient cell lines (Figure 6A and B). As has been seen with other Chk1 inhibitors, the most robust potentiation was observed with gemcitabine across the range of cell lines. For gemcitabine, not only did V158411 reduce the EC_50_ of the chemotherapeutic agent but it also increased the fraction of cells killed. In the ovarian carcinoma cell line SKOV-3, V158411 modestly potentiated the cytotoxic activity of carboplatin and cisplatin but not oxaliplatin (Figure 6C). Western blotting analysis revealed that all three platinum drugs increased the phosphorylation of Chk1 on Ser296 but only the combination of cisplatin with V158411 robustly induced H2AX phosphorylation on Ser139 (Figure 6D). As well as exhibiting single agent activity against TNBC and ovarian cancer cell lines, V158411 potentiated the cytotoxicity of chemotherapeutic drugs in these tumor types suggesting that Chk1 inhibitors either alone or in combination could be a viable treatment option in these tumor types.

## Discussion

Multiple Chk1 inhibitors are currently undergoing clinical testing in combination with a variety of cytotoxic chemotherapeutic agents for their ability to potentiate the anti-tumor efficacy of the chemotherapy drugs whilst not increasing the systemic toxicity of these drugs. Recent work using loss-of-function siRNA screens or small molecule Chk1 inhibitors have begun to identify tumor types where Chk1 is critical for cancer cell proliferation and/or viability in the absence of a DNA damaging chemotherapeutic agent. To date, neuroblastoma [33], melanoma [34], Myc driven lymphomas [35, 36], leukemia and lymphoma cell lines [37, 38] and Fanconi’s Anemia cells [29] have been identified to be Chk1 kinase dependent. This suggests that there may be subsets of cancers for which a Chk1 inhibitor, administered as a single agent, could be a useful therapeutic option.

We postulated that cancers with underlying defects in DNA repair, DNA damage response or DNA replication may be suitable candidates for single agent Chk1 inhibitor therapy. Spontaneous triple-negative breast cancer shares many of the characteristics of tumors derived from patients carrying mutations in the BRCA gene. BRCA is known to be involved in a variety of DNA repair pathways such as homologous recombination and base excision repair and are exquisitely sensitive to inhibitors of poly (ADP-ribose) polymerase. Recent reports have demonstrated the sensitivity of TNBC to PARP inhibitors as well as DNA damaging cytotoxic agents such as gemcitabine and cisplatin [14]. TNBCs have been shown to have reduced expression of DNA repair genes involved in base excision repair, nucleotide excision repair and the Fanconi’s Anemia repair pathways [39]. This suggests that like cancers that arise in BRCA mutation carriers, spontaneous TNBCs may harbor underlying defects in DNA repair and we hypothesized that this cancer sub-type may be a suitable candidate for single agent Chk1 inhibitor therapy.

This hypothesis was substantiated in a screen of 26 solid cancer cell lines where TNBC cancer cell lines were among the most sensitive to growth inhibition by the novel, selective Chk1 inhibitor V158411. Even though V158411 is an extremely selective inhibitor of Chk1, it is difficult to ascertain the absolute selectivity of any small molecule kinase inhibitor. To confirm this observation, two structurally unrelated Chk1 kinase inhibitors PF-477736 [26] and AZD7762 [27] potently inhibited the proliferation of TNBC breast cancer cell lines compared to two ER-positive cell lines suggesting that the anti-proliferative effects observed were due to Chk1 inhibition and not the inhibition of an off target kinase. However, the sensitivity to the Chk1 inhibitors was not just limited to the TNBC cell lines as the HER2-postive, ER-negative SKBr3 breast cancer cell line and the SKOV-3 ovarian cancer cell line (HER2-positve, ER-positive but estrogen insensitive [40]) were among the most sensitive cell lines to Chk1 inhibitor induced cell death. These results have recently been confirmed by another study demonstrating that four TNBC cell lines (including HCC1937, MDA-MB-157 and MDA-MB-468 used in this study) had reduced viability following Chk1 knockdown with a specific siRNA [41]. Additional studies have demonstrated that the Chk1 inhibitor AZD7762 synergistically combined with numerous PARP1 inhibitors (including olaparib, rucaparib or ABT888) to inhibit the growth of mammary carcinoma cells *in vitro* and *in vivo* [42, 43]. In these studies, AZD7762 demonstrated little single agent activity in the breast cancer cell lines at the concentration tested. Of the cell lines tested in these papers, only two overlapped with our study.

In light of these results, we attempted to understand the mechanism by which single agent Chk1 inhibitors induced TNBC and ovarian cancer cell death. The clearest marker of response to Chk1 inhibition in all the sensitive cell lines was the time dependent increase in phosphorylation of H2AX on serine 139. Phosphorylation on this site is generally associated with an increase in DNA double strand breaks [44]. Coupled with Chk1 inhibition reducing cell viability and inducing caspase-3/7 dependent apoptosis and DNA fragmentation, cell death following Chk1 inhibition appears to be most likely via increased DNA double strand breaks. The mechanism for the generation of these breaks is not completely clear. However, since Chk1 inhibition caused a dramatic decrease in the fraction of cells in G1 that were unable to complete S-phase and accumulate in mitosis suggests that replication fork collapse and the subsequent formation of DSBs by the DNA endonuclease Mus81/Eme1 [45] is responsible.

In all breast and ovarian cancer cell lines, including those relatively resistant to V158411 single agent cytotoxicity, reduction in total Chk1 protein levels following V158411 treatment was evident. This was especially notable at the higher concentrations of V158411 and appeared to correlate with an increase in pH2AX (S139) in the V158411 sensitive cell lines. V158411 treatment of leukemia and lymphoma cell lines induced Chk1 degradation that was proteasome dependent [38] whilst degradation of Chk1 was observed in HT29 cells following treatment with four structurally distinct Chk1 inhibitors in combination with gemcitabine: V158411, LY2603618, MK-8776 and GNE-900 [46]. Chk1 is deactivated through protein degradation in response to replication and genotoxic stress. Phosphorylation of Chk1 at serine 317 and 345 by ATR promotes Chk1 activation but also induces the ubiquitin-proteasome dependent degradation of Chk1 [47–49]. Given that V158411 induces phosphorylation of Chk1 at serine 317 and 345 in breast and ovarian cancer cells, this degradation of Chk1 reflects the normal homeostatic mechanism of checkpoint resetting.

The precise mechanism for the sensitivity of the TNBC and ovarian cancer cell lines compared to other solid cancer cell lines remains to be fully understood. The resistance of the two ER-positive breast cancer cell lines BT474 and MCF-7 could not be overcome with 4-hydroxytamoxfien suggesting that estrogen receptor signaling did not contribute to the relative sensitivities in the breast cancer cell lines. Chk1 expression has been demonstrated to be elevated in histological grade 3 TNBC primary tumors compared to other grade 3 breast cancers [50]. Sensitivity of the TNBC and ovarian cancer cell lines did not correlate with total Chk1 protein expression levels but did correlate closely with the levels of phosphorylation of Chk1 on serine 296 and to a lesser extent serine 317 but not with serine 345. This observation matched that of Cole *et al.* [33] who identified neuroblastoma as a potential therapeutic target for Chk1 inhibition and that sensitivity to Chk1 inhibition by either siRNA or small molecules correlated with Chk1 S296 phosphorylation. The HER2 positive breast cancer cell line SKBr3 was extremely sensitive to growth inhibition by all three Chk1 inhibitors tested. Whilst this cell line had low levels of Chk1 phosphorylation at serine 296, it was the only cell line with high expression of pH2AX (S139) potentially indicative of a reasonable level of DNA breakage in proliferating cells. Shibata *et al.*, [51] identified elevated expression levels of pChk1 (S317) and to a lesser extent pH2AX (S139) as being predictive of the sensitivity of breast cancer cell lines to the Chk1 inhibitor PF-477736. Sensitivity to V158411 appeared independent of both p53 and *kRas* mutational status, both of which have previously been implicated in Chk1’s mechanism of action [52]. The outlier in this analysis was the ovarian cancer cell line ES-2. This cell line had high expression levels of pChk1 (S296) but was relatively resistant to growth inhibition by all three Chk1 inhibitors. Further work is needed to understand the relative resistance of this cell line to Chk1 inhibition.

The underlying mechanism for the sensitivity of these cancer cell types to single Chk1 inhibitor therapy is not yet clear and the phosphorylation events identified as potential predictive markers of sensitivity (pChk1 (S296), (S317) and pH2AX (S139)) are most likely symptomatic rather than the cause of the underlying sensitivity. This observation suggests that the molecular defects in these cell lines occur in pathways for which Chk1 can mutually compensate to protect genomic integrity and therefore Chk1 inhibition is lethal. An example of this so far discovered is the Fanconi’s Anemia (FA) DNA repair pathway. Cells defective in FA were sensitive to Chk1 siRNA and the small molecule Go6976 due to an accumulation of unrepairable DNA double strand breaks [29]. The basal like breast cancer cell line HCC9137 harbors a homozygous truncation mutation in the DNA repair gene *BRCA1* [53] and this reduced capacity to repair DNA breaks may underlie this cell lines sensitivity. Underlying defects in DNA repair would be predicted to confer increased sensitivity to DNA damaging cytotoxic drugs such as cisplatin. The correlation between sensitivity to cisplatin and V158411 was cell line dependent and not consistent across the panel of breast and ovarian lines studied. For example, the BRCA defective cell line HCC1937 was highly sensitive to cisplatin and V158411 whilst the ovarian cell line SKOV-3 was equally sensitive to V158411 but 8-fold more resistant to cisplatin than the HCC1937 cell line. This suggests that different mechanisms may account for the V158411 sensitivity in different cell lines.

Cells under replicative stress due to oncogene amplification or activation of oncogenic signaling pathways are addicted to Chk1 kinase activity for the completion of a normal S-phase [35, 36]. The sensitivity of neuroblastoma and melanoma cell lines has been suggested to be likely related to oncogenic replicative stress. In the case of neuroblastoma, this has been linked to members of the Myc family of oncogenes [33, 34]. Therefore the sensitivity of the breast and ovarian cell lines in this study could be attributed to underlying defects in DNA repair or DNA damage signaling pathways, oncogene induced replicative stress or a combination of the two. Chk1 has been demonstrated to be important for replication origin firing, high rates of replication fork progression and replication fork stabilization [34, 54, 55]]. Inhibition of Chk1 may result in increased replication origin firing and reduced fork progression leading to increased regions of RPA bound ssDNA. This in turn leads to ATR activation and tumor cell apoptosis.

Triple-negative status was not a sufficient prognostic marker for response to Chk1 inhibition as other cell line types such as the HER2 positive SKBr3 breast cancer cell line and SKOV-3 ovarian cancer cell line were among the most sensitive cell lines to growth inhibition by V158411. For this treatment to be better focused in the clinic to those patients most likely to benefit then additional biomarkers prognostic of sensitivity would be extremely beneficial. High expression of pChk1 (S296) in untreated tumors and the induction of pH2AX (S139) post Chk1 inhibitor therapy appear to be the most sensitive and predictive markers for response to Chk1 inhibitor therapy. However, phospho-protein profiling of tumor biopsies by immunohistochemistry or western blotting is not without its technical challenges. Alternative methods of identifying potential clinical responders, such as gene signature profiles, would aid the use of Chk1 inhibitors as monotherapy.

From this study and others, it is becoming clear that Chk1 inhibitors may have clinical utility as a single agent as well as in combination with cytotoxic chemotherapy agents in a variety of human cancer types. Chk1 inhibitors either as single agents or in combination with cytotoxic chemotherapy are a potentially viable therapeutic option for the treatment of triple-negative breast cancer in the clinic. High tumor expression of pChk1 (S296) could serve as a useful biomarker to select those patients who would most likely benefit from Chk1 inhibitor therapy.

## Conclusions

Clinical testing of Chk1 inhibitors is currently focused on their ability to potentiate the anti-tumor efficacy of cytotoxic chemotherapy drugs and anti-metabolite therapies. Recent studies have demonstrated single agent activity of Chk1 inhibitors in cancer cells harboring defects in DNA damage repair or response pathways or high levels of replicative stress. V158411 either as a single agent or in combination with cytotoxic chemotherapy are potentially viable therapeutic options for the treatment of triple-negative breast cancer in the clinic. High tumor expression of pChk1 (S296) could serve as a useful biomarker to select those patients who would most likely benefit from Chk1 inhibitor therapy. The BRCAness of triple-negative breast cancer may underlie the sensitivity of this cancer type to Chk1 inhibition.
